# The role of structural parameters in DNA cyclization

**DOI:** 10.1186/s12859-016-0897-9

**Published:** 2016-02-04

**Authors:** Ludmil B. Alexandrov, Alan R. Bishop, Kim Ø. Rasmussen, Boian S. Alexandrov

**Affiliations:** Theoretical Division, Los Alamos National Laboratory, Los Alamos, NM USA; Center for Nonlinear Studies, Los Alamos National Laboratory, Los Alamos, NM USA

## Abstract

**Background:**

The intrinsic bendability of DNA plays an important role with relevance for myriad of essential cellular mechanisms. The flexibility of a DNA fragment can be experimentally and computationally examined by its propensity for cyclization, quantified by the Jacobson-Stockmayer J factor. In this study, we use a well-established coarse-grained three-dimensional model of DNA and seven distinct sets of experimentally and computationally derived conformational parameters of the double helix to evaluate the role of structural parameters in calculating DNA cyclization.

**Results:**

We calculate the cyclization rates of 86 DNA sequences with previously measured J factors and lengths between 57 and 325 bp as well as of 20,000 randomly generated DNA sequences with lengths between 350 and 4000 bp. Our comparison with experimental data is complemented with analysis of simulated data.

**Conclusions:**

Our data demonstrate that all sets of parameters yield very similar results for longer DNA fragments, regardless of the nucleotide sequence, which are in agreement with experimental measurements. However, for DNA fragments shorter than 100 bp, all sets of parameters performed poorly yielding results with several orders of magnitude difference from the experimental measurements. Our data show that DNA cyclization rates calculated using conformational parameters based on nucleosome packaging data are most similar to the experimental measurements. Overall, our study provides a comprehensive large-scale assessment of the role of structural parameters in calculating DNA cyclization rates.

**Electronic supplementary material:**

The online version of this article (doi:10.1186/s12859-016-0897-9) contains supplementary material, which is available to authorized users.

## Background

From a physical perspective the DNA molecule is a long polymer chain [1, 2]. The inherent sequence specific flexibility of this biopolymer is essential for its ability to support tissue-specific cellular functionality [3, 4], by permitting it to alter its conformation, e.g., for binding of transcription factors to DNA [5–9]. Due to the semi-flexibility of the double helix it has typically been modeled as an elastic rod with mechanical properties well described by the wormlike chain model (WLC) [10].

In the basic WLC model, the conformational properties of double-stranded DNA depend solely on its persistence length, which is approximately 150 base pairs (bp) [11, 12]. Within this model, any DNA loops and sharp bends shorter than the persistent length are energetically costly and the probability for their spontaneous creation is negligibly small [13]. Therefore, the basic WLC model predicts that the probability for cyclization, quantified in terms of the Jacobson-Stockmayer J factor [14, 15], for a contact to occur between two ends of a DNA polymer shorter that 150 bp is vanishingly small. This prediction is in contrast to various in vitro [16] and in vivo [17] observations. A multitude of different experiments (for example, measurements using ligase proteins [16], small angle x-ray scattering coupled with atomic force microscopy [18], etc.) have provided evidence for significantly larger cyclization probabilities (J factors) than the ones predicted by the basic WLC model. While there have been some arguments about details in the earlier experiments [19], a recent study based on single-molecule fluorescence resonance has also demonstrated a high cyclization of short DNA fragments (shorter than 70 bp) on a single-molecule level [20].

Evidently, the basic WLC model consistently describes the cyclization of long DNA fragments, while it is generally unable to accurately evaluate the cyclization of ultra-short DNA fragments [20–22]. This is perhaps unsurprising given that the basic WLC model examines DNA as a uniform biopolymer, while ignoring both its three-dimensional (3D) molecular structure and its nucleotide sequence; both of which may significantly affect the cyclization rate. For example, by ignoring the 3D nature of DNA, the basic WLC model does not account for the requirement of proper torsional orientation of DNA fragments and cannot describe the experimentally observed oscillations of DNA cyclization rates that results from the natural 10 base pair torsional period of the molecule [23]. Additionally, previous experimental studies have shown that periodic stretches of consecutive adenine-thymine base pairs demonstrate curved equilibrium conformations [24]. These periodic DNA sequences possess natural sequence dependent static bending (also known as intrinsic curvature), which is not taken into account by the basic WLC model and can lead to a higher cyclization rate [25, 26].

Accounting for the three-dimensional structure and sequence dependent static bending of DNA allows a better representation of the true elastic nature of the double helix [27]. As a first approximation, the static bending (although statistical in its nature) can be considered as an equilibrium property of each DNA fragment [28]. To calculate more realistically cyclization properties of DNA, a coarse-grained Monte Carlo approach incorporating the three-dimensional structure and intrinsic curvature of DNA was proposed by Levenet, Crothers, and Zhang [29, 30] and by Czapla, Swigon, and Olson (referred to as the CSO model in the text) [31]. In accordance with the Cambridge convention for DNA conformation [32], this approach describes the relative orientation and displacement of successive DNA base pairs by six helicoidal structural parameters: helix twist angle, roll angle, tilt angle, shift displacement, slide displacement, and rise displacement. To account for thermal fluctuations, these conformational parameters are considered to be given by normal distributions with specific expectations values and standard deviations. The expectation values define the static bending, while the standard deviations define the flexibility and depend on DNA’s elastic moduli. Thus, in the CSO model, the curvature of a DNA fragment depends both on the nucleotide sequence of the fragment and the expectation values of these conformational parameters. Further, each (random) configuration of a DNA fragment depends on the deviations from the expectation values (caused by fluctuations), which are governed by the DNA elastic moduli (see Methods for more details).

Previous work using the CSO model [31] has demonstrated that taking into account the three-dimensional structure and intrinsic curvature of a short DNA fragment allows better estimates of its cyclization that are more compatible with the measurements from experimental studies [16]. These previous applications of the CSO model relied on expectation values of the conformational parameters that are generated by considering DNA as either a homogenous ideally straight fragment or a periodic curved fragment. Importantly, based on the idea that the physicochemical properties of DNA play an important role in protein-DNA interaction [33–35], and this approach recently shed light on the interplay between DNA flexibility and protein binding [7, 36].

In this study, we evaluate the role of static bending in computationally determined DNA cyclization rates by applying the CSO model with several sets of expectation values of the conformational parameters. These sets of values were obtained from different experimental studies and/or computational analyses (Table 1). It should be noted that previous studies [37, 38] have performed some comparison between computationally predicted and experimentally measured cyclization factors. The focus of these early studies was on 11 sequences with lengths between 150 and 160 bp, whereas, here we examine approximately eight times more experimentally measured sequences with lengths between 57 and 325 bp as well as of 20,000 randomly generated DNA sequences with lengths between 350 and 4000 bp. More specifically, we first validate our implementation of the CSO model by comparing it to previous analyses using the artificial expectation values for both ideally straight and curved DNA fragments [31]. Next, we curated the literature for different sets of conformational parameters as well as for DNA sequences with experimentally measured cyclization rates. In total, we curated seven distinct sets of conformational parameters as well as 86 DNA sequences with experimentally measured cyclization factors and lengths between 57 and 325 bp. For each set of conformational parameters, we calculated the cyclization rates of the 86 curated DNA sequences and compared the *in silico* obtained cyclization rates with the experimental measurements. Our analyses show that DNA cyclization rates calculated using conformational parameters based on nucleosome packaging data [39, 40] are most similar to the experimental measurements. Our results also demonstrate that none of the examined sets of conformational parameters accurately describe cyclization of DNA fragments with lengths less than 100 bp. Lastly, we calculated the cyclization of 20,000 randomly generated DNA sequences with lengths between 350 and 4000 bp using each of the seven distinct sets of conformational parameters. Our data demonstrate that, for these 20,000 random sequences, all sets of parameters yield very similar results comparable to the experimentally measured cyclization rates. In summary, this study provides a comprehensive examination of the role of static bending, represented by various sets of experimentally measured or calculated structural parameters, in computationally estimating DNA cyclization.

**Table 1:** List of used sets of structural parameters

Parameter set name	Reference	Derivation approach	Number of nucleotides	Tilt	Roll	Twist	Shift	Slide	Rise
SET1	(Zhou et al., 2013) [43]	All-atom Monte Carlo simulations	Pentanucleotides	No	Yes	Yes	No	No	No
SET2	(Gabrielian and Pongor, 1996) [45]	Computationally combining SET3 and SET4	Trinucleotides	No	Yes	No	No	No	No
SET3	(Brukner et al., 1995) [44]	Endonuclease experiments	Trinucleotides	No	Yes	No	No	No	No
SET4	(Goodsell and Dickerson, 1994) [40]	Nucleosome positioning	Trinucleotides	No	Yes	No	No	No	No
SET5	(Ulyanov and James, 1995) [46]	NMR spectroscopy	Dinucleotides	Yes	Yes	Yes	Yes	Yes	Yes
SET6	(Rachofsky et al., 2001) [47]	Computational analysis of X-ray crystallography	Dinucleotides	Yes	Yes	Yes	Yes	Yes	Yes
SET7	(Olson et al., 1998) [48]	Computational analysis of X-ray crystallography	Dinucleotides	Yes	Yes	Yes	Yes	Yes	Yes

Reference information, derivation approach, and number of nucleotides are provided for each set of parameters. Additionally, the table denotes with “Yes” which of the six types of parameters is provided in the respective references. “No” is equivalent to using a default value for all nucleotide combinations. Default values: tilt = 0.00°; helix twist = 34.30°; shift = 0.00 Å; slide = 0.00 Å; rise = 3.40 Å. The exact values for each set of parameters are provided in Additional file 1: Tables S3 through S9 respectively for SET1 through SET7

## Results and discussion

We developed a computational implementation of the original CSO model and perform large amounts of simulations (generating a total of ~10^18^ chain representations). To verify our implementation, we first used two sets of artificial expectation values for the DNA conformational parameters given in the original CSO paper: (i) the set of parameters in which DNA is assumed to be a homogenous and ideally straight sequence and (ii) the set of parameters in which DNA is assumed to be a periodic curved sequence. These two sets of parameters were used in a previous study [31] to estimate the cyclization rates for DNA fragments with various lengths. Similarly to the original application of the CSO model, we assumed that the fluctuations of the tilt and roll angles are exactly the same (i.e., isotropic bending) and for the root-mean-square fluctuations we used the value of 4.84°, which corresponds to a persistence length of ~147 bp. Furthermore, we used the previously proposed value of 4.09° for the root-mean-square fluctuations in the helix twist, which corresponds to a global twisting constant compatible with previously measured equilibrium topoisomer distributions of DNA mini-circles [41, 42]. Most theoretical and experimental studies report DNA cyclization of a given sequence in terms of the Jacobson-Stockmayer J factor, which represent the ratio of the equilibrium constants for cyclization to the bimolecular association of a linear molecule [14]. The J factor reflects the efficiency of fragment cyclization and it can be experimentally measured as well as computationally calculated using various methodologies (Methods).

The model of a straight homogenous DNA sequence corresponds essentially to a straight elastic rod. In this case we use for equilibrium helix twist of 34.28° while the equilibrium rise displacement is set at 3.40 Å for all possible dinucleotides (Additional file 1: Table S1). All other conformational parameters (angles as well as displacements) are set to zero. Using our implementation of the CSO model, we simulated DNA fragments with lengths between 110 and 400 base pairs based on the conformational parameters of the straight model. Our simulations yielded J factors that matched the previously reported values [31] (Additional file 2: Figure S1).

In the model of curved DNA, the molecule is considered to possess a sequence that naturally curves the molecule to a nearly circular configuration for 150 bp long fragments. This model introduces sequence dependence as it considers two distinct, albeit artificial, types of nucleotides: X and Z. For this model, conformational parameters are considered based on dinucleotides (Additional file 1: Table S2). The XX and XZ base steps have a helix twist of 36.00° (effectively resulting in one complete turn per 10 base pairs) and roll and tilt angles of zero degrees. In contrast, ZZ and ZX base steps have a slightly lower helix twist of 35.57°, roll angle of 7.41°, and tilt angle of 0.00°. The conformational displacements for all dinucleotides are the same as in the straight model (Additional file 1: Table S2). Using our implementation of the CSO model, we simulated DNA fragments with lengths between 70 and 180 base pairs based on the conformational parameters of the curved model. Similarly to the straight model, our simulations yielded J factors that were the same as the ones previously reported in ref. [31] (Additional file 3: Figure S2).

### Curation of DNA conformational parameters

We examined the literature to curate previously reported DNA conformational\structural parameters. In total, we were able to identify seven distinct sets of parameters generated by various experimental methodologies and/or theoretical approaches. For simplicity, we have termed these sets of parameters SET1 through SET7 and provided summary information about each set of parameters in Table 1. Furthermore, the actual values of these sets of parameters are provided also as Additional file 1: Tables S3–S9. Briefly, SET1 provides conformational parameters for each pentanucleotide sequence, which were calculated by leveraging all-atom Monte Carlo simulations and further validated by X-ray crystallography, NMR spectroscopy, and hydroxyl radical cleavage data combined with statistical analysis and molecular dynamics simulations [43]. SET2, SET3, and SET4 provide conformational parameters for each trinucleotide sequence. The conformational parameters in SET4 are derived based on nucleosome packaging data [39, 40], while the ones in SET3 are based on endonuclease experimental data [44]. SET2 was previously generated as a combination of the two other trinucleotide sets of conformational parameters [45]. SET5, SET6, and SET7 provide equilibrium structural parameters for each dinucleotide sequence and were generated, respectively, by NMR spectroscopy [46], and two different computational analysis of X-ray crystallography [47, 48]. It should be noted that for some sets of parameters only some of the six parameters types were available (Table 1). For example, for SET4, information was provided only for the roll angles between dinucleotides but not for any other parameter. In such cases, we used as default values the following conformational parameters: tilt = 0.00°; helix twist = 34.30°; shift = 0.00 Å; slide = 0.00 Å; rise = 3.40 Å.

### Curation of DNA sequences with experimentally measured cyclization factors

Experimentally measuring the J factor of a DNA fragment is complicated and time-consuming process that requires significant efforts even for a single sequence. Thus, it is not surprising that the amount of available DNA fragments with experimentally characterized J factors is limited. Overall, our curation of DNA sequences resulted in identifying 86 DNA sequences previously reported in [6, 20, 21, 49, 50]. While we were able to identify additional studies that have experimentally characterized J factors of DNA fragments, these reports lacked details needed for our analysis. Most commonly, the exact DNA nucleotide sequence of the reported DNA fragments was not given. Fragments without exact information about their DNA nucleotide sequences were excluded from our analyses as such fragments can be only examined using sequence independent parameter sets (for example, when DNA is considered as homogenously straight or ideally curved) and such examinations have already been performed by others [31]. To facilitate future examination of DNA J factors, we have provided all curated information (including DNA sequences and experimentally measured J factors) as Additional file 1: Table S10.

### Comparing experimentally measured J factors with *in silico* calculations

**Fig. 1:**
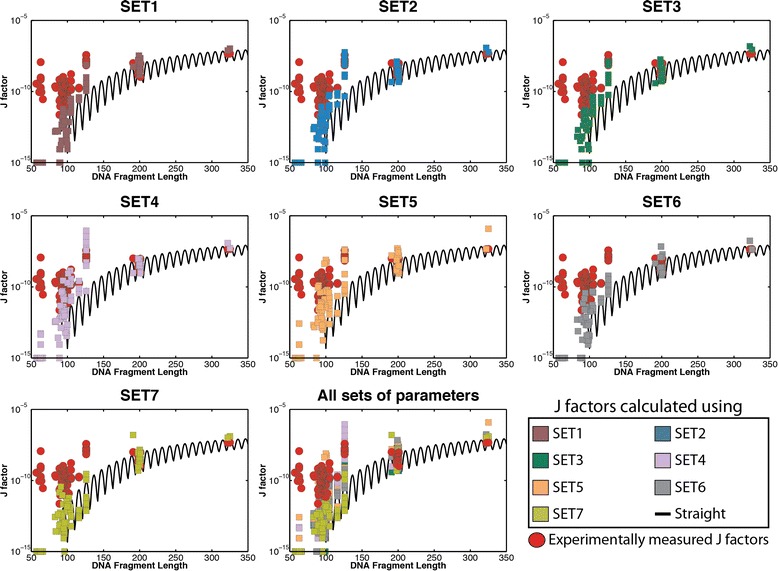
Comparison between computationally estimated J factors and experimentally derived J factors. A panel is provided for each of the seven sets of curated structural parameters (Table 1). In each panel, the computationally estimated J factors of 86 DNA fragments are plotted using *filled squares* with a color reflecting the set of structural parameters that was used to derive them. In all panels, the experimentally measured J factors for the same 86 DNA sequences are shown as *red circles*. All *horizontal axes* are depicted using the same scale and reflect the length of the plotted DNA fragments. Similarly, all *vertical axes* are shown using identical logarithmic scales and reflect the values of either computationally estimated or experimentally derived J factors. The *black line* in all panels reflects J factors estimated based on the model of straight DNA. For clarity, computationally estimated J factors with values lower than 10^−15^ are shown as 10^−15^

We applied our implementation of the CSO model to each of the DNA fragments with experimentally measured J factors. For each DNA sequence, we independently performed simulations with each of the seven sets of curated conformational\structural parameters and calculated the respective J factors (Fig. 1). In all simulations we used 4.84° for root-mean-square fluctuations in both tilt and roll angles, while 4.09° is used for the root-mean-square fluctuations of helix twist. The *in silico* calculated J factors for each sequence are provided in Additional file 1: Table S10. Visual comparison reveals an overlap between *in silico* calculated J factors and their experimental counterparts when examining sequences with lengths longer than 100 bp, for most sets of conformational parameters (Fig. 1). In contrast, regardless of the used set of conformational parameters, the *in silico* calculated J factors for sequences with lengths less than 100 bp were, for almost all examined sequences, orders of magnitude lower than the experimental measurements (Fig. 1).

**Fig. 2:**
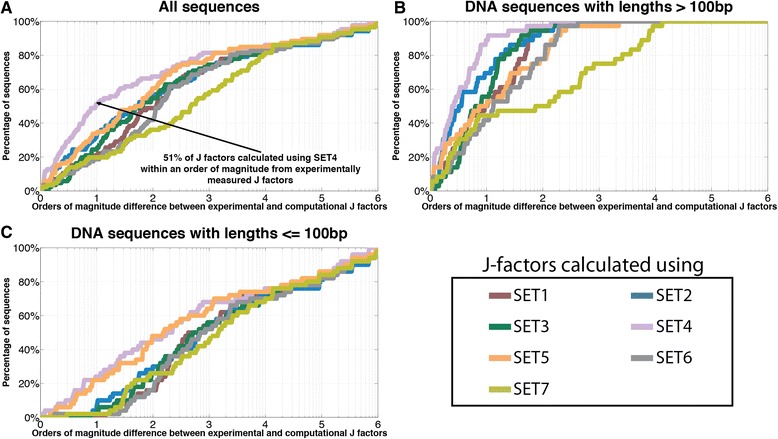
Evaluating seven sets of curated structural parameters for accurately estimating experimental J factors. Each of the three panels contains seven different curves with colors corresponding to the respective set of structural parameters. The y-axes reflect the orders of magnitude difference between experimentally measured and computationally derived J factors. The x-axes correspond to the percentage of sequences for a given order of magnitude difference. (**a**) Curves based on all examined DNA sequences; (**b**) Curves based on DNA sequences with lengths longer than 100 bp; (**c**) Curves based on DNA sequences with lengths shorter than or equal to 100 bp

To quantify the differences between *in silico* calculated J factors and the experimental measurements, we calculated the percentage of sequences for which their computationally estimated J factors are within a particular absolute distance from their actual experimentally measured J factors (Fig. 2). For example, for SET4, 51 % of examined sequences have *in silico* calculated J factors within an order of magnitude from the respective experimental measurements (Fig. 2a). Comparing the percentage operator curves for all examined sequences reveals that two sets of conformational parameters yield results most similar to the experimental measurements: SET4 and SET5. Stratifying the DNA fragments based on their lengths (Fig. 2b and c) reveals that SET4 outperforms all other sets of conformational parameters for sequences longer than 100 bp (Fig. 2b). Both SET4 and SET5 yield similar results and, hence, computational J factors most similar to the experimental measurements for sequences shorter than 100 bp (Fig. 2c).

Interestingly, our analysis reveals a significant discrepancy in the ability of the CSO model to accurately estimate J factors for sequences with lengths less than 100 bp even for the best performing set of equilibrium parameters (Fig. 2b and c). For SET4, 89 % of the *in silico* calculated J factors where within an order of magnitude of their experimental measurements for sequences longer than 100 bp (Fig. 2c). This percentage drops to 24 % for sequences shorter than 100 bp (Fig. 2b). These results emphasize the need for developing more elaborate models that can better explain cyclization of ultra-short DNA fragments.

Lastly, for some sequences longer than 200 bp, calculating J factors with different sets of conformational parameters yields significantly different results. For example, the estimated J factor of sequence CA_325bp (a DNA segment with a length of 325 bp) calculated using SET5 is almost 30 times higher when compared to the J factor of the same sequence calculated using SET6 (Additional file 1: Table S10). This fact demonstrates that the choice of conformational parameters can affect strongly the calculation of a J factor even for longer sequences. Further, using simulated nucleotide sequence data, we will explore the dependence between sets of conformational parameters and computationally estimating a J factor of sequences longer than 350 bp.

### Evaluating J factors of randomly generated DNA fragments longer than 350 bp

Our curated set of DNA fragments with experimentally characterized J factors did not contain any sequences longer than 350 bp. To address this limitation, we generated 20,000 random sequences with lengths between 350 and 4000 bp. These sequences were divided into groups of 1000 sequences (i.e., 1000 random sequences each with length of 350 bp; 1000 random sequences each with length of 400 bp; …; 1000 random sequences each with length of 4000 bp). We calculated the J factors for all 20,000 random sequences using each of the seven sets of curated conformational parameters.

**Fig. 3:**
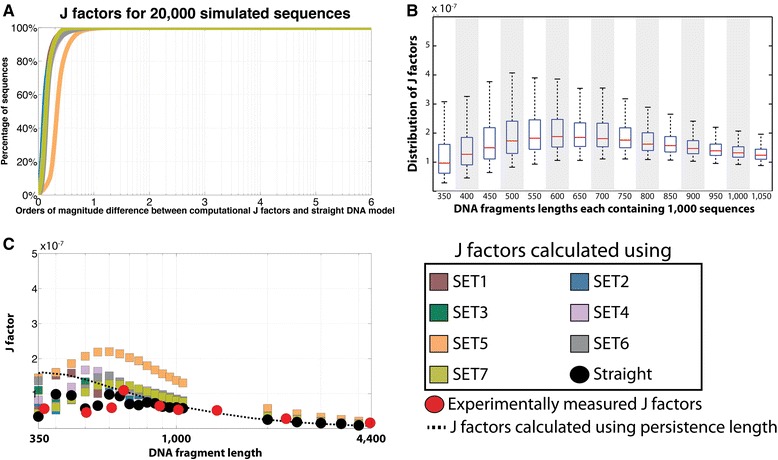
Evaluating J factors of *in silico* generated DNA fragments. **a** Seven different curves with colors corresponding to the respective set of structural parameters are shown. Each curve reflects the analysis of 20,000 random sequences with lengths between 350 and 4000 bp. The y-axis reflects the orders of magnitude difference between computationally derived J factors and the straight DNA model. The x-axis corresponds to the percentage of sequences for a given order of magnitude difference. **b** Distributions of J factors for 15,000 random sequences calculated using SET7 with lengths between 350 and 1050 bp. The plot is stratified for the different DNA fragment lengths. *Red lines* reflect median values, while the *blue box* shows the 25 and 75 % quantiles. **c** Average J factors for 20,000 simulated sequences and seven sets of structural parameters are plotted as *squares* in colors corresponding to the respective set of structural parameters. J factors calculated based on the straight DNA model [31] are shown as *black circles* and ones based on DNA persistent length [55] are depicted as a *dotted line*. Lastly, *red dots* are used to display ten experimentally measured J factors for long DNA sequences as reported in [21]

Comparing the differences between the J factors of straight homogeneous DNA sequences calculated with different sets of parameters revealed that the CSO model yields very similar results for each of the seven sets of conformational\structural parameters (Fig. 3a). The only exception is SET5 (Fig. 3a). The discrepancy in performance for SET5 is consistent with the previous observation that this set of parameters performs poorly for longer DNA fragments (Fig. 2b and c). Nevertheless, our results demonstrate that for sequences longer than 350 bp the *in silico* derived J factors are mostly independent of the choice of conformational parameters and that these J factors are consistent with the results obtained with the parameters for straight DNA.

Examining the distributions of J factors calculated using SET7 (note that other sets of equilibrium parameters yield very similar results for long sequences, with the exception of SET5) reveals that, even for the 1000 random sequences each with length of 350 bp, all *in silico* calculated J factors are within an order of magnitude of one another (Fig. 3b). Furthermore, longer random sequences have very similar J factors close to experimental observations (Fig. 3c) [21], indicating that the nucleotide structure of a DNA fragment plays a less significant role for estimating the cyclization factors of longer DNA sequences (Fig. 3b and c). Our examination of 20,000 randomly generated sequences with different lengths revealed that J factors of long DNA fragments, estimated using the CSO model, are generally independent of the choice of conformational parameters or from the nucleotide structure (Fig. 3c). For such sequences, using the conformational parameters of straight DNA allows accurate evaluation of fragment cyclization rate.

## Conclusions

In this study, we applied the CSO model [31] to perform a large-scale examination of the effect that different structural/conformation parameters have on estimating J factors of DNA sequences with different lengths. We applied our implementation of the CSO model to 86 DNA fragments with experimentally characterized J factors, with lengths between 57 and 325 bp, as well as to 20,000 *in silico* generated random sequences, with lengths between 350 and 4000 bp. Our analysis demonstrates that SET4 provides results most similar to the experimental measurements. Nevertheless, we show that even this set of parameters performs poorly for DNA fragments shorter than 100 bp. The analysis of J factors calculated for the *in silico* generated DNA sequences indicates that for sequences longer than 350 bp the choice of structural parameters and the nucleotide sequence of a DNA fragment makes little difference in estimating the cyclization of that fragment.

The superior performance of the CSO model with SET4 over the other sets of parameters is somewhat unexpected. This parameter set is based on an examination of nucleosome positioning performed almost 30 years ago [39, 40] and it only provides information about one of the six structural parameters, viz., the roll angle (Table 1; Additional file 1: Table S6). In contrast, some of the other sets of parameters were generated using more recent experimental/theoretical approaches and provide information about all six helicoidal structural parameters (Table 1). One plausible, albeit speculative, explanation of SET4’s performance is that the intrinsic bending propensities of the curated sequences closely resemble the ones of nucleosome sequences, thus, allowing for SET4 to best describe the cyclization of the examined DNA fragments.

Our analyses also revealed significant differences (up to six orders of magnitude) between experimentally measured and theoretically estimated J factors for some of the examined short sequences regardless of the set of parameters. One possible reason for this observation is that none of the parameter sets is sufficiently accurate for describing such sequences. A more likely explanation is that the CSO model fails to capture the salient physics at the short segment lengths and needs to be further elaborated to accurately describe the cyclization of such sequences. In support of the latter explanation, it was previously suggested that (especially for fragments shorter than 70 bp) there is a need to consider a kink-able WLC [22, 51] and/or melt-able WLC [4, 52, 53] models. Future studies will be needed to evaluate the performance of such models in regards to a large collection of DNA fragments with experimentally measured J factors.

Finally, there are a number of confounding factors that might be affecting our analyses and subsequent results. Our examination relies on seven curated sets of parameters. For each set of parameters, the (sequence-dependent) mean values of the six types of helical structural/conformational parameters were used for estimating cyclization of DNA fragments. These experimentally and computationally derived values demonstrate large standard deviations (for example, see ref. [48]). This heterogeneity was ignored by our analysis and the mean values, given in the corresponding articles, were assumed to be both representative and generalizable. Nevertheless, assuming the *bona fide* nature of the experimentally measured J factors as well as the mean values of the curated structural parameters, this study provides a comprehensive large-scale evaluation of the role of structural parameters in calculating DNA cyclization rates.

## Methods

### Theoretical framework underlying the CSO model

In the CSO model [31], each random configuration of a DNA segment depends on its sequence via the equilibrium (minimum energy) values of the conformational parameters, helix twist angle, roll angle, tilt angle, shift displacement, slide displacement, and rise displacement, Θ^0^ = (*θ*_*k*1_^0^; *θ*_*k*2_^0^; *θ*_*k*3_^0^; *θ*_*k*4_^0^; *θ*_*k*5_^0^; *θ*_*k*6_^0^) for each (*k*^th^) base pair defined in relation to the previous (k-1)^th^ base pair. The deviations from these equilibrium values is caused by thermal fluctuations and controlled by the elastic moduli, *f*_*ij*_^*k*^ (where, *f*_*ij*_^*k*^ are the elements of the symmetric 6 × 6 elastic force matrix *F*, normalized to the thermal energy, *βF*). The energy of each base pair, in harmonic approximation depends only on the deviations, ΔΘ, from the expectation values of the parameters. The total energy of a DNA sequence is the sum of the energies over all, *N*, base pairs of the sequence.

In the CSO model, the energy for each consecutive base pairs, (k-1, k), in harmonic approximation, is given by:

$$ {G}_k\left(\Theta, F,{\Theta}^0\right)=\frac{1}{2}{\displaystyle \sum_{i=1}^6}{\displaystyle \sum_{j=1}^6}{f}_{ij}^k\left({\theta}_{ki}-{\theta}_{ki}^0\right)\left({\theta}_{kj}-{\theta}_{kj}^0\right)\equiv \frac{1}{2}{\displaystyle \sum_{i=1}^6}{\displaystyle \sum_{j=1}^6}{f}_{ij}^k\Delta {\theta}_{ki}\Delta {\theta}_{kj} $$

Thus, for each generated random DNA configuration the total energy is simply the sum of the energies over all consecutive base pairs:

$$ G\left(\Theta, F,{\Theta}^0\right)={\displaystyle \sum_{k=1}^N}{G}_k\left(\Theta, F,{\Theta}^0\right)=\frac{1}{2}{\displaystyle \sum_{k=1}^N}{\displaystyle \sum_{i=1}^6}{\displaystyle \sum_{j=1}^6}{f}_{ij}^k\Delta {\theta}_{ki}\Delta {\theta}_{kj}. $$

Therefore, the probability, *P*, for each set of consecutive base pairs (k-1, k) to be in a given configuration, defined by the structural parameters Θ, is related to the temperature, T, by the Boltzmann factor via:

$$ {\mathrm{P}}_k\left(\Theta \right)\sim {e}^{-\frac{G_k\left(\Theta, F,{\Theta}^0\right)}{kT}}\equiv {e}^{-\frac{\beta }{2}{\displaystyle \sum_{i=1}^6}{\displaystyle \sum_{j=1}^6}{f}_{ij}^k\Delta {\theta}_{ki}\Delta {\theta}_{kj}}, $$

where $\upbeta =\frac{1}{kT}$, and *k* is the Boltzmann constant. Further, if one performs diagonalization of the force-constant matrix *F* and rewrites the energy for each consecutive base pairs in terms of a diagonal matrix *D* and normal variables ω_,_${G}_k=\frac{1}{2}{\Omega}^TD\Omega$, the probability for a given configuration becomes

$$ {\mathrm{P}}_k\left(\Theta \right)={\displaystyle \prod_{i=1}^6}\frac{1}{\sqrt{2\pi \beta {D}_{ii}}}{e}^{-\frac{\beta }{2}{D}_{ii}{\omega}_k^2} $$

Thus, we can represent the probability as a product of independent terms and normal variables that describe the changes of the parameters on the directions of the principal axes of deformation.

### *In silico* estimation of J factors using the CSO model

Monte Carlo simulations have been the preferred method for estimating the propensity for DNA cyclization within the CSO model. The calculations sample the configuration space of the chains by generating series of DNA sequences with random structural parameters, distributed normally with given expectation values and standard deviations. It should be noted that calculating the J factor of a short DNA segment is computationally very expensive since such J factors are usually between 10^−8^ and 10^−14^, thus requiring large Monte Carlo sampling (usually between 10^12^ and 10^16^ DNA configurations). To make the simulations feasible we utilize the half-chain sampling enhancement technique proposed by Alexandrowicz [54]. Following earlier works [29–31] the J factor can be presented, as a product of probabilities describing the contribution of the spatial configuration:

$$ J=\frac{4\pi }{N_A}\ W\left(r\approx 0\right){\Gamma}_r\left( cos\gamma \approx 1\right){\Phi}_{r,\  cos\gamma}\left(\phi \approx 0\right) $$

Here, *W*(*r* ≈ 0) is the probability for a DNA segment to be circular, i.e., to posses the end-to-end distance *r* ≈ 0. In practice, a threshold of 30 Å was used as previously done in [31]. The factor Γ_*r*_(*cosγ* ≈ 1) is the conditional probability that the normal of the first and last base pairs are (almost) aligned when the ends of the fragments coincide, i.e., the cosine of the net bending angle is ≈ 1. In practice, a threshold of *cosγ* > 0.86 was used when *r* ≈ 0 as previously done in [31]. The term Φ_*r*, *cosγ*_(*ϕ* ≈ 0) is the conditional probability that the first and last base pairs coincide, i.e., the helix twist angle is approximately zero. In practice, a threshold of *cosϕ* > 0.86 was applied when both *r* ≈ 0 and the first and the last base pairs are coplanar, i.e., *cosγ* ≈ 1. The factor $\frac{4\pi }{N_A}$, where *N*_*A*_ is the Avogadro’s number, is the normalizations associated with the uniformly distributed probability density of bimolecular association.

### Experimentally estimating the J factor of a DNA sequence

Experimentally, the cyclization propensity of a DNA fragment is characterized by the ratio of the equilibrium constants for cyclization versus bimolecular association of a linear DNA molecule [14]. This ratio is usually referred to as the J factor of a DNA segment and experimentally measured by the formula:

$$ J=2{M}_0\underset{t\to 0}{ \lim}\frac{C(t)}{D(t)}, $$

here M_0_ is the starting (t = 0) concentration of initial DNA fragments, *C(t)* is the concentration of the monomeric (fixed by ligation) circular species, and *D(t)* is the concentration of the dimeric species (i.e., bimolecular reaction via the sticky ends of the initial fragments).

### Curating conformational parameters and experimentally measured J factors

Curation was performed by examined the previously published literature and all curated data is provided in the Additional file 1: Table S10. Curating experimentally derived J factors was focused on studies where both the experimental measurements and the DNA fragments’ nucleotide sequences were provided. Curating conformational parameters focused only on sequence dependent (i.e., dinucleotides, trinucleotides, etc.) of at least one of the six types of helicoidal structural parameters. It should be noted that for certain studies, which did not provide numeric values for some of their measurements but rather plotted their data, we digitalized the provided figures to extract the necessary information.

### Computational implementation of the CSO model and generation of random sequences

A novel computational implementation of the hitherto described model was developed and validated (see Results). For simplicity, an illustrative working MATLAB implementation of the code is provided for the model of straight DNA in Additional file 4. For each DNA fragment, the code was run until the error for calculating the J factor was less than 5 % or until 5 × 10^15^ chains were generated for that fragment. Fragments that had no circular configurations after calculating 5 × 10^15^ trajectories are reported with a J factor of 10^−15^.

Distinct random DNA sequences were generated assuming equal probability for each DNA nucleotide.

## Additional files

Additional file 1: Table S1. Structural parameters for the straight homogenous DNA model. **Table S2**: Structural parameters for the curved DNA model. **Table S3**: Structural parameters for SET1. **Table S4**: Structural parameters for SET2. **Table S5**: Structural parameters for SET3. **Table S6**: Structural parameters for SET4. **Table S7**: Structural parameters for SET5. **Table S8**: Structural parameters for SET6. **Table S9**: Structural parameters for SET7. **Table S10**: Sequences with experimentally measured and computational calculated J factors. (XLSX 127 kb) Additional file 2: Figure S1. Validation of the CSO implementation using the model of a straight homogenous DNA sequence. (PDF 178 kb) Additional file 3: Figure S2. Validation of the CSO implementation using the model of a curved DNA sequence. (PDF 194 kb) Additional file 4:
**Illustrative working MATLAB implementation of the code for the model of straight DNA.** (PDF 214 kb)
