# Supplementary material for: The role of structural parameters in DNA cyclization
Source: BMC Bioinformatics. 2016 Feb 4;17:68. doi: 10.1186/s12859-016-0897-9 (PMC4743258; doi:10.1186/s12859-016-0897-9)
Supplement: Additional file 2: Figure S1. — Validation of the CSO implementation using the model of a straight homogenous DNA sequence. (PDF 178 kb) [file 12859_2016_897_MOESM2_ESM.pdf]

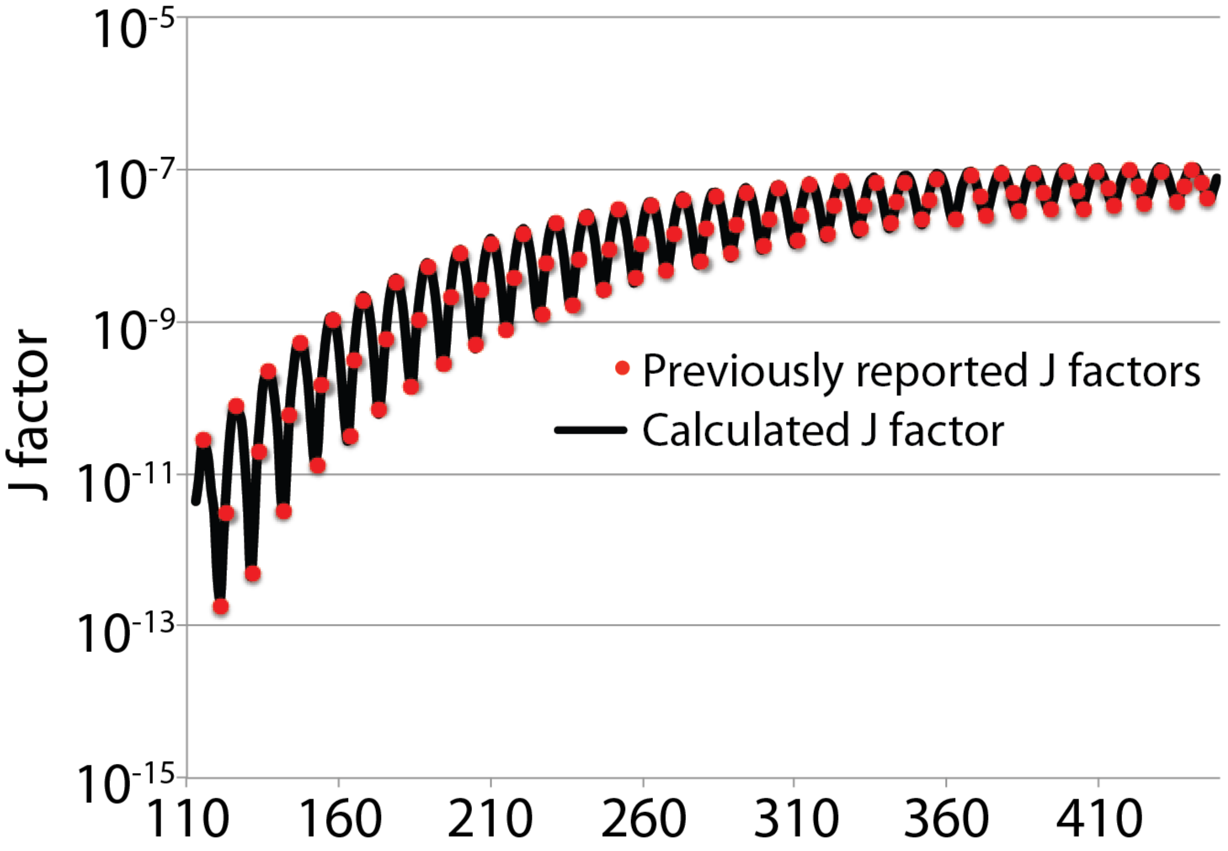

S1. Figure

Figure S1. Validation of the CSO implementation using the model of a straight homogenous DNA sequence. Black line corresponds to calculated J factors, while red circles are used to show J factors based on results reported in ref. (29). The Y-axis depicts J factors (log scaled), while the X-axis reflects the length of the DNA fragment.
