# Supplementary material for: The role of structural parameters in DNA cyclization
Source: BMC Bioinformatics. 2016 Feb 4;17:68. doi: 10.1186/s12859-016-0897-9 (PMC4743258; doi:10.1186/s12859-016-0897-9)
Supplement: Additional file 4: — Illustrative working MATLAB implementation of the code for the model of straight DNA. (PDF 214 kb) [file 12859_2016_897_MOESM4_ESM.pdf]

Additional file 4. Illustrative working MATLAB implementation of the code for the model of straight DNA.

```
%% Starting a clean session without any variables and initiating random number generator
clear all;
clc;
rng(1234613717); % init random generator with the same seed

%% Model parameters for a mononucleotide sequence (i.e., XXXXX...)
% Average values (in radians and nanometers)
avg_tilt   = 0.000000000; % 0.000000000 [radians]  ==  0.000000000 [degrees]
avg_roll   = 0.000000000; % 0.000000000 [radians]  ==  0.000000000 [degrees]
avg_twist  = 0.598298867; % 0.598298867 [radians]  == 34.280000000 [degrees]

avg_shift  = 0.000000000; % 0.000000000 [angstrom] == 0.000000000 [nanometers]
avg_slide  = 0.000000000; % 0.000000000 [angstrom] == 0.000000000 [nanometers]
avg_rise   = 3.400000000; % 3.400000000 [angstrom] == 0.340000000 [nanometers]

% Standard deviations (in radians and nanometers)
std_tilt   = 0.084473935; % 0.084473935 [radians]  == 4.840000000 [degrees]
std_roll   = 0.084473935; % 0.084473935 [radians]  == 4.840000000 [degrees]
std_twist  = 0.071383966; % 0.071383966 [radians]  == 4.090000000 [degrees]

std_shift  = 0.141421356; % 0.141421356 [angstrom] == 0.0141421356 [nanometers]
std_slide  = 0.141421356; % 0.141421356 [angstrom] == 0.0141421356 [nanometers]
std_rise   = 0.141421356; % 0.141421356 [angstrom] == 0.0141421356 [nanometers]

% Sequence params and tresholds for hits
Q = 0.7604; % normalization factor
base_pairs   = 211;          % integer number of XXXXX… bases (mononucleotide sequence)
tresh_r      = 30.0000000000;  % 30.0000000000 [angstrom] == 3.0000000000 [nanometers]
tresh_deg30  = 0.86602540378;  % cos(30 [degrees]) = sqrt(3)/2 = 0.86602540378

%% Performing Monte Carlo calculations
Pr  = 0; Ptr = 0; Ptw = 0; % initializing hit counts

for MC_iter = 1 : 5*10^15 %  performing 5*10^15 Monte Carlo iterations

    for base_index = 1 : base_pairs % looping the basepairs in the sequence

     % Generating normally distributed random numbers with normrnd
     % normrnd is a MATLAB program, where normrnd(mu,sigma)
     % generates random numbers from the normal distribution
     % with mean parameter mu and standard deviation parameter sigma
     tilt_angle   = normrnd( avg_tilt,  std_tilt  );
     roll_angle   = normrnd( avg_roll,  std_roll  );
     twist_angle  = normrnd( avg_twist, std_twist );

     shift   = normrnd( avg_shift, std_shift );
     slide   = normrnd( avg_slide, std_slide );
     rise    = normrnd( avg_rise,  std_rise  );

     % Angle calculations
     if ( (tilt_angle==0) && ( roll_angle == 0 ) ) % MATLAB cannot handle atan2(zero divided by zero)
         phase_angle = 0.0000000000;
     else
         phase_angle = atan2( tilt_angle / roll_angle );
     end

     net_bend_angle = sqrt( tilt_angle^2 + roll_angle^2 );
```

```
    % Matrix and vector calculations
    % roty and rotz are MATLAB functions for rotation matrices along y and z, respectively
    Tn      = rotz( twist_angle/2 - phase_angle ) * roty( net_bend_angle )   * rotz( twist_angle/2 + phase_angle );
    Tn_half  = rotz( twist_angle/2 - phase_angle ) * roty( net_bend_angle/2 ) * rotz( phase_angle );
    rn      = Tn_half * [shift; slide; rise];

    if ( base_index == 1 ) % first base in the sequence: init An
      An   = [ Tn, rn; 0 0 0 1];
    else % next bases in the sequence: mutiply An to the previous values
      An   = An * [ Tn, rn; 0 0 0 1];
    end

   end

   if ( sqrt(An(1,4)^2 + An(2,4)^2 + An(3,4)^2) <  tresh_r )
    Pr = Pr + 1;

    if ( abs( An(3,3)) > tresh_deg30 )
      Ptr = Ptr + 1;

      Tn = An(1:3, 1:3);
      trace_Tn = trace(Tn);

      if ( abs( (trace_Tn - Tn(3,3)) / (1 + Tn(3,3)) ) > tresh_deg30 )
        Ptw = Ptw + 1;
      end
    end

   end

end

Jfactor = Ptw / (5*10^15 * Q);
```
